# Discovery of Quality Markers in Hugan Qingzhi Formula by Integrating a Lipid-Lowering Bioassay with UHPLC-QQQ-MS/MS

**DOI:** 10.1155/2020/1594350

**Published:** 2020-11-12

**Authors:** Fan He, Chun-Xin Xiao, Can-Jian Wang, Jie Liang, Qi-Qing Cheng, Li Zhang, Ben-Jie Zhou, Hua Zhou

**Affiliations:** ^1^State Key Laboratory of Quality Research in Chinese Medicine and Faculty of Chinese Medicine, Macau University of Science and Technology, Taipa, Macao, China; ^2^Department of Pharmacy, The Seventh Affiliated Hospital, Sun Yat-sen University, Shenzhen, China; ^3^Zhuhai Hospital of Integrated Traditional Chinese and Western Medicine, Zhuhai City, Guangdong Province 519000, China; ^4^Joint Laboratory for Translational Cancer Research of Chinese Medicine of the Ministry of Education of the People'sRepublic of China, Macau University of Science and Technology, Taipa, Macao, China

## Abstract

Nonalcoholic fatty liver disease (NAFLD) is a prevalent chronic liver disease. The Hugan Qingzhi formula (HGQZ) has been proven effective in treating NAFLD through clinical and pharmacological mechanism studies. A screening study of the chemical components was carried out to better control the quality of this formula. Current research has combined biological activity assessment with chemical analysis to screen and identify the bioactive compounds in HGQZ for use as potential quality markers (Q-markers) to control the quality of this herbal product. The HGQZ extracted by three different solvents was evaluated in a free fatty acid-induced hepatic steatosis LO2 cell model. Simultaneously, the twelve major chemical constituents of these extracts were quantitatively measured by ultrahigh-performance liquid chromatography coupled with triple quadrupole mass spectrometry (UHPLC-QQQ-MS/MS). Extraction with 50% ethanol showed the most potent lipid-lowering effect in steatosis LO2 cells and the highest extraction rate of major chemical constituents. Correlation analysis was used to establish the relationship between the biological activities and chemical characteristics of these extracts. The results showed that the contents of typhaneoside, hyperoside, isoquercitrin, isorhamnetin-3-O-neohesperidoside, notoginsenoside R1, and alisol B 23-acetate were positively correlated to the lipid-lowering effect. The subsequent bioassay confirmed that typhaneoside, isoquercitrin, and alisol B 23-acetate played the role of reducing the lipid effect. In conclusion, 50% of ethanol extraction produced the most active extract of HGQZ. Typhaneoside, isoquercitrin, and alisol B 23-acetate could be considered potential Q-markers for the quality control of HGQZ.

## 1. Introduction

Nonalcoholic fatty liver disease (NAFLD) is one of the most common chronic liver diseases in humans [1]. This disease is characterized by excessive hepatic fat accumulation due to significant ethanol depletion and viral infection [2, 3]. In Western countries, the prevalence of NAFLD is between 17% and 33% and significantly increases to 80% in obese individuals [4]. However, there is no ideal method to control NAFLD.

The Hugan Qingzhi formula (HGQZ) is an effective traditional Chinese herbal medicine (CHM) for treating NAFLD based on the clinical experience of Dr. Kuntang Zang, a prestigious traditional Chinese medicine (TCM) doctor at the Southern Medical University of China. HGQZ is formulated with five herbs, including *Alismatis Rhizoma* (AR), *Crataegi Fructus* (CF), *Nelumbinis Folium* (NF), *Typhae pollen* (TP), and *Notoginseng Radix* (NR). Previous studies have shown that HGQZ is effective for treating NAFLD in *in vivo* and *in vitro* models [5, 6]. Mechanistic studies have shown that HGQZ acts on adenosine monophosphate-activated protein kinase (AMPK), mitogen-activated protein kinase (MAPK), and peroxisome proliferator-activated receptor (PPAR) signaling pathways to produce therapeutic effects on NAFLD [7–9]. The main constituents of HGQZ include triterpenes, flavonoids, glycosides, alkaloids, saponins, and organic acids. Previously, the lipid-lowering effect of three compounds, i.e., alisol A 24-acetate (derived from AR), ursolic acid (derived from CF), and nuciferine (derived from NF), had been proved in *in vitro* studies [10]. Thus, it is unknown whether these compounds are suitable to indicate the quality of HGQZ. In addition, the relationship between the bioactivity and the content of these marker compounds has not yet been established in HGQZ. Therefore, it is very important to investigate the relationship between the bioactivity and chemical constituents of HGQZ and discover more bioactive compounds that can be used for the quality control of HGQZ.

Currently, a method commonly used to quantitatively control CHM's quality is to measure the content of one compound or several compounds at most in a CHM. The compound used for quality control is usually highly abundant in the CHM but not necessarily bioactive nor essential to the function of the CHM, and therefore, it is called a marker compound or index component. This method is derived from natural drug chemistry and takes the quality control system of synthetic drugs as a reference. The shortcoming of this kind of quality control method is evident because the ability of CHM to treat diseases is based on multiple components in CHM being directed at multiple targets. Therefore, an ideal quality control marker for CHM should reflect this attribute of CHM, and thus, the concept of quality markers (Q-markers) was proposed in 2016 by Prof. Liu et al., an academician of the Chinese Academy of Engineering [11]. This concept considered the complex chemical composition and the TCM theory, thus enhancing quality control in terms of the consistency, controllability, and traceability of CHM [12]. We have made several attempts to develop holistic strategies for achieving CHM quality control. The criteria of Q-markers were based on combinational methods consisting of bioactivity assays, chemical profiling, network pharmacological analysis, and multivariate statistical methods [13–19]. The markers discovered by these methods showed the features of Q-markers and were more relevant to the functions of CHM. Therefore, Q-markers should be generally applied to the quality research of herbal medicines.

In the present study, the bioactivities of different extracts of HGQZ were first compared. Ultrahigh-performance liquid chromatography coupled with triple quadrupole mass spectrometry (UHPLC-QQQ-MS/MS) was applied for the qualification of twelve major chemical components of HGQZ, i.e., epicatechin, nuciferine, typhaneoside, rutin, isorhamnetin-3-O-neohesperidoside, hyperoside, isoquercitrin, notoginsenoside R1, quercetin, isorhamnetin, alisol A 24-acetate, and alisol B 23-acetate. The Pearson correlation analysis was used to analyze the relationship between chemical markers and pharmacological effects to predict the potential Q-markers of HGQZ. Finally, the predicted Q-makers were confirmed by *in vitro* bioassays.

## 2. Materials and Methods

### 2.1. Sample Preparation

The samples of AR (batch no.: 20160801), FC (batch no.: 161208), PN (batch no.: 161104), PT (batch no.: 160701), and RN (batch no.: 161212) were collected from their respective Good Agricultural Practice for Chinese Crude Drugs (GAP) bases in Fujian, Shandong, Anhui, and Yunnan Provinces. They were authenticated by Chun-Song Cheng, a researcher working at Macau University of Science and Technology, based on the requirements of these herbs according to the Chinese Pharmacopeia [20]. Voucher specimens were stored in the State Key Laboratory of Quality Research in Chinese Medicine. The prescription of HGQZ was composed of AR (15 g), CF (15 g), NF (10 g), TP (7.5 g), and NR (2.5 g). All herbs were pulverized and passed through a 60-mesh sieve. Raw materials were weighed according to their proportions in the prescription to obtain fifty grams in total per sample. Sample one was refluxed three times with six volumes (v/w) of water, each time for one hour, to obtain the water extract (WE) of HGQZ. Sample two was refluxed similarly with 50% ethanol to obtain the ethanol extract (EE). Sample three was fluxed twice with six volumes of water for one hour. Then, it was refluxed further with 50% ethanol using the same procedure. The mixture combining with these two extracts was the water plus ethanol extract (WEE). The three extracts were filtered, spray-dried at 60°C, and dried in a vacuum oven at 60°C until a constant weight was obtained. This research used the dried extracts in powder form.

For the bioactivity assay, the extracts were dissolved in DMSO and diluted with culture medium to the indicated concentrations calculated against herbal raw materials. For example, 1.5 mg/mL means that 1 mL of the extract was extracted from 1.5 mg of raw material. The final concentration of DMSO was 0.1%.

### 2.2. UHPLC-QQQ-MS/MS Analysis

#### 2.2.1. Preparation of Standard Solutions

**Figure 1:**
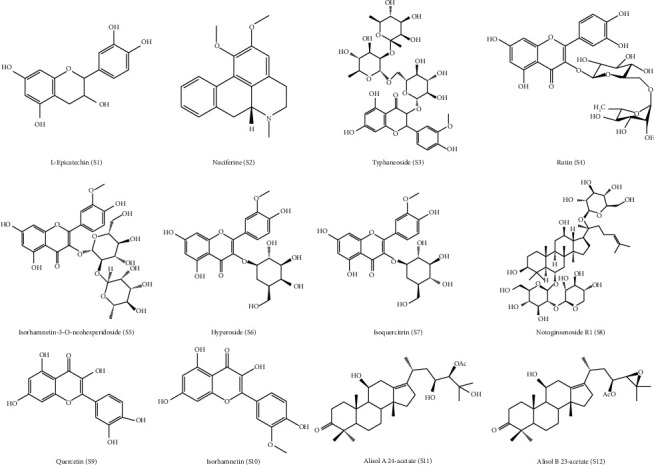
The chemical structures of the 12 standard reference compounds in HGQZ.

Epicatechin (S1), nuciferine (S2), typhaneoside (S3), rutin (S4), isorhamnetin-3-O-neohesperidoside (S5), hyperoside (S6), isoquercitrin (S7), notoginsenoside R1 (S8), quercetin (S9), isorhamnetin (S10), alisol A 24-acetate (S11), and alisol B 23-acetate (S12) were purchased from the National Institutes for Food and Drug Control (Beijing, China) as chemical reference standards (approximately 98% purity, quantification grade). The chemical structures are shown in Figure 1. Purified water was produced by a Milli-Q system (Millipore, Milford, MA, USA). Acetonitrile was of HPLC grade, and the other chemical reagents were of analytical grade. Twelve standard products were accurately weighed and dissolved by methanol to prepare a stock solution. The stock solution of the 12 compounds was prepared in a suitable ratio to obtain a standard mixed solution. The concentrations were 1.15 (S1), 1.11 (S2), 1.36 (S3), 1.23 (S4), 1.38 (S5), 0.97 (S6), 1.21 (S7), 1.38 (S8), 1.57 (S9), 1.02 (S10), 1.30 (S11), and 1.10 mg/mL (S12).

#### 2.2.2. Apparatus and Conditions

An Agilent 1290 series UHPLC system (Agilent Technologies, Santa Clara, CA) with a Waters Acquity UPLC C_18_ column (2.1 mm × 100 mm, 1.7 *μ*m) (Milford, MA, USA) was used for analysis. The mobile phases were introduced by gradient elution; mobile phase A was 0.1% formic acid and water, and mobile phase B was acetonitrile. The gradient program was as follows: 0–6 min, 18–20% B; 6–7 min, 20–25% B; 7–7.01 min, 25–50% B; 7.01–10 min, 50–90% B; and 10–12 min, 90% B. The flow rate was 0.35 mL/min, the injection volume was 2 *μ*L, and the column temperature was 30°C. The mass spectrometric analysis was carried out with an Agilent 6460 QQQ-MS system (Agilent Technologies, Santa Clara, CA, USA) equipped with an electrospray ionization (ESI) source operated in positive and negative ionization modes. The 12 compounds were injected into the MS system to obtain MS information.

The multiple reaction monitoring (MRM) parameters fragmentor voltage (FV) and collision energy (CE) were as follows to detect the 12 compounds: *m/z* 289.1⟶245.1 (FV, 130; CE, 10) for S1, *m/z* 296.2⟶265.2 (FV, 100; CE, 14) for S2, *m/z* 769.2⟶314.0 (FV, 270; CE, 42) for S3, *m/z* 609.3⟶300.1 (FV, 230; CE, 34) for S4, *m/z* 623.3⟶314.1 (FV, 210; CE, 30) for S5, *m/z* 463.2⟶300.0 (FV, 170; CE, 22) for S6 and S7 (FV, 190; CE, 26), *m/z* 955.3⟶775.2 (FV, 280; CE, 46) for S8, *m/z* 301.0⟶151.0 (FV, 140; CE, 18) for S9, *m/z* 315.1⟶300.2 (FV, 130; CE, 18) for S10, *m/z* 577.4⟶531.4 (FV, 140; CE, 10) for S11, and *m/z* 515.4⟶107.1 (FV, 140; CE, 98) for S12. The optimal MS parameters were a drying gas (N_2_) flow rate of 11.0 L/min, drying gas temperature of 300°C, a nebulizer pressure of 15 psig, and a capillary voltage of 4000 V. Our previous study showed this method to be sensitive, rapid, and accurate.

#### 2.2.3. Sample Preparation and Analysis

Two hundred milligrams of each extract were accurately weighed, added to 50 mL of 70% methanol (v/v), and ultrasonically extracted for 30 min; then, the extract was filtered, and the resulting filtrate was collected. One milliliter of the resulting filtrate was accurately drawn into a 5 mL volumetric flask and diluted to volume with 70% methanol to prepare the test solution. The solution was filtered with a 0.22 *μ*m microporous membrane and analyzed with UHPLC-QQQ-MS/MS.

#### 2.2.4. Method Validation

The method was validated by following the guidelines for the validation of quality standard of TCM from the Pharmacopoeia of the People's Republic of China (2015 Edition, Volume 4).

The calibration curve was constructed by determining the relationship between the peak area and the concentration for at least six different concentration levels. The linearity of each compound in the studied concentration (*R*) range was good (*r* > 0.999). The lower limit of quantification (LLOQ) is defined as the lowest concentration that can be determined with a signal-to-noise ratio of 10 : 1. The limit of detection (LOD) is the concentration with a signal-to-noise ratio of 3 : 1. The precision was measured by analyzing a standard solution mixed continuously six times a day. The RSDs of the results were less than 5.0%. The stability was determined by testing a test solution stored at room temperature repeatedly at 0, 2, 4, 6, 8, and 12 h in one day. The RSD of the peak area of each compound should not be larger than 5%. The repeatability was determined by testing a sample that was separated into six portions and extracted separately. The RSD of the content level of each compound should not be larger than 5%. The recovery was determined by spiking a certain amount (approximately 0.1 g) of a sample extract with a known amount of the mixed standards repeatedly six times. The recovery rates of the 12 compounds should be within the range from 95% to 105%, and the RSD of the recovery rate of each compound should not be larger than 5%.

### 2.3. Cell Viability Assay

LO2 cells were purchased from China Cell Culture Center (Shanghai, China). They were cultured in RPMI-1640 medium (10% FBS, 100 U/ml penicillin, and 0.1 mg/ml streptomycin) at 37°C under 5% CO_2_. LO2 cells were seeded in each well of a 96-well plate at 5 × 10^3^/well and incubated for 24 h. The cells were treated with WE, WEE, and EE at 94, 187, 365, 750, and 1500 *μ*g/mL with or without free fatty acid (FFA, 2 : 1 oleic acid: palmitic acid ratio) at a concentration of 1 mm. The cultured medium was discarded after 24 h. Ten microliter MTT assay reagents (5 mg/mL) and 100 *μ*L fresh medium were added to each well. The absorbance was measured at 570 nm by a SpectraMax Paradigm Multimode Microplate Reader (Molecular Devices, LLC, Sunnyvale, CA, USA).

### 2.4. Establishment of the Hepatic Steatosis Model and Treatment

The hepatic steatosis model was established in LO2 cells according to reported methods [21]. In brief, the cells were treated for 24 h with 1 mM FFA consisting of oleic acid and palmitic acid in a 2 : 1 ratio. Unless otherwise specified, the treatments were divided into the control group (normal LO2 cells), FFA group (LO2 cells treated with FFA alone), WE group (LO2 cells treated with FFA and WE at 750 *μ*g/mL), WEE group (LO2 cells treated with FFA and WEE at 750 *μ*g/mL), EE group (LO2 cells treated with FFA and EE at 750 *μ*g/mL), and positive group (LO2 cells treated with FFA and alisol A 24-acetate at 10 *μ*M).

### 2.5. Nile Red Staining for Lipid Measurement

The cells were stained with Nile red (1 *μ*g/mL) after washing with phosphate-buffered saline (PBS). The SpectraMax Paradigm Multimode Microplate Reader (Molecular Devices, LLC, Sunnyvale, CA, USA) was used to quantify the fluorescence response at a wavelength of 510 nm for excitation and 580 nm for emission to determine the lipid content.

### 2.6. Measurement of Total Cholesterol and Triglyceride

According to the instruction of the manufacturer, the content of total cholesterol (TC) and triglyceride (TG) was measured using commercial kits purchased from Nanjing Jiancheng Bioengineering Institute Co, Ltd (Nanjing, China). Cell lysates (from 1 × 10^6^/dishes) were prepared on ice using a 1% radioimmunoprecipitation assay (RIPA) buffer. The protein concentrations were determined with a BCA protein assay kit (http://corporate.thermofisher.com/en/contact.html, Waltham, MA, USA). The results of TC and TG were expressed as mg/mg protein and mmol/g protein, respectively.

### 2.7. Potential Quality Marker Prediction Based on Pearson Correlation Analysis

Pearson correlation analysis was used to determine the relationship between the lipid-lowering effect and the content of components in the three extracts. A component with a correlation coefficient >0.970 was considered a potential Q-marker. Statistical analysis and analysis of variance (ANOVA) were performed with GraphPad Prism 6 (GraphPad Software, La Jolla, CA, USA), and a two-sided *P*value of <0.05 was considered significant.

### 2.8. Verification of Quality Markers in Free Fatty Acid-Induced LO2 Cells

Six monomers, including typhaneoside, hyperoside, isoquercitrin, isorhamnetin-3-O-neohesperidoside, notoginsenoside R1, alisol B 23-acetate, and alisol A 24-acetate (positive group), were separately dissolved in DMSO to achieve final concentrations of 10 mM. The cells were grouped as the control group, FFA group, FFA added typhaneoside (10 *μ*M) group, FFA added hyperoside (10 *μ*M) group, FFA added isoquercitrin (10 *μ*M) group, FFA added isorhamnetin-3-O-neohesperidoside (10 *μ*M) group, FFA added notoginsenoside R1 (10 *μ*M) group, FFA added alisol B 23-acetate (10 *μ*M) group, and FFA added alisol A 24-acetate group (10 *μ*M). The final concentration of FFA was 1 mM for every group. After 24 h, TC was measured according to the method described in Section 2.5. Each treatment was performed in triplicate.

## 3. Results

### 3.1. Optimization of UPLC-MS/MS Conditions

Acetonitrile and water (0.1% formic acid) were selected as the mobile phases because of the high resolution and short analysis time achieved with acetonitrile, and 0.1% formic acid was added to improve the ionization response and inhibit peak tailing. Due to the diversity of compounds in the formula, it was difficult to analyze all compounds in a single MS detection mode. Therefore, negative ion mode was used to detect the flavonoids, and positive ion mode was used to detect the alkaloids and saponins.

**Figure 2:**
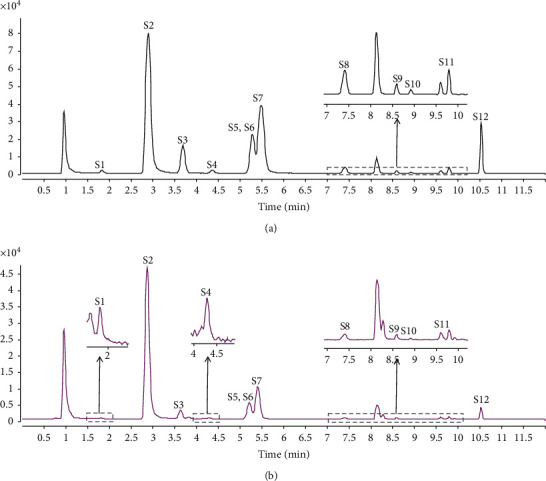
Representative MRM chromatograms of the standard mixture (a) and sample (b). (S1, L-epicatechin; S2, nuciferine; S3, typhaneoside; S4, rutin; S5, isorhamnetin-3-O-neohesperidoside; S6, hyperoside; S7, isoquercitrin; S8, notoginsenoside R1; S9, quercetin; S10, isorhamnetin; S11, alisol A 24-acetate; S12, alisol B 23-acetate).

The parameters of the fragmentation energy and impact voltage were optimized to obtain the most abundant relative abundance under the MRM conditions. The results are shown in Figure S1 (supplementary material). The MRM chromatograms of the standard solution and sample solutions are shown in Figure 2, which indicates that the chromatographic peaks were well separated under the above conditions. No interference peaks were found at the same retention time of the standard materials.

The linear parameters used for the target compounds are listed in Table 1. The linearity of each compound in the studied concentration range was good (*r* > 0.999). The linear range satisfied the content of the 12 components in the samples. The LLOQs and LODs for the 12 components are listed in Table 1. The RSDs of the precision, stability, and repeatability determined for quantitating the 12 compounds were less than 5.0% (Table 1). The recovery was verified by adding known amounts of standards into the HGQZ extract. The recovery varied from 97.53% to 101.14% with RSDs between 1.15% and 3.77% (Table 1). All the results of the detection method met the requirements of methodological verification.

### 3.2. Quantification of the 12 Compounds in Different Extracts of HGQZ

Sample quantification was conducted to uncover the chemical profiles of HGQZ. In this study, the 12 active components of the five herbs in HGQZ used to treat NSFLD were selected as Q-markers of the HGQZ formula. Quantitative analysis was carried out by using the standard external method. The contents of the compounds analyzed are listed in Table 2. The results showed that the extracts contained high contents of isoquercitrin, typhaneoside, isorhamnetin-3-O-neohesperidoside, and notoginsenoside R1 (Table 2). In addition, extraction with 50% ethanol yielded the highest extraction rate of the major chemical components, followed by WEE and WE.

### 3.3. Effects of Different Extracts on Cell Viability in LO2 Cells

Cell viability assays were performed to assess the cytotoxicity of WE, WEE, and EE. LO2 cells were treated with WE, WEE, and EE (94, 187, 365, 750, and 1500 *μ*g/mL) in the absence or presence of FFAs for 24 h, and the viability was determined by MTT assays. The results showed that three extracts did not significantly affect the cell viability of normal LO2 cells at the indicated concentrations (Figures S2(a)–S2(c)). WE and WEE exhibited no cytotoxicity in FFA-induced LO2 cells (Figures S2(d) and S2(e)). However, EE significantly decreased cell viability at a concentration of 1500 *μ*g/mL (Figure S2(f), *P* < 0.01). Therefore, the highest dose of extracts used in further experiments was 750 *µ*g/mL.

### 3.4. Effects of Different Extracts on FFA-Induced Lipid Accumulation and Total Glycerol Enhancement

**Figure 3:**
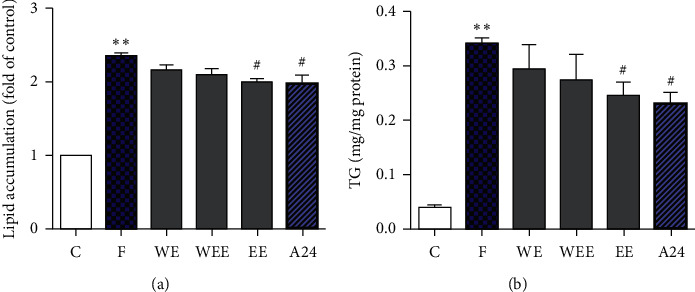
The effect of different fractions on lipid accumulation and total glycerol (TG) in the free fatty acid- (FFA-) induced LO2 cell model. C, control group; F, FFA group; WE, FFA and water extract (750 *μ*g/mL) group; WEE, FFA and water and ethanol double extract (750 *μ*g/mL) group; EE, FFA and 50% ethanol extract (750 *μ*g/mL) group; A24, FFA and alisol A 24-acetate (10 *μ*m) group. The final concentration of FFA in each group was 1 mM. Data are presented as the mean ± SD of three independent experiments. *n* = 3. ^∗∗^*P* < 0.01 vs. control group; ^#^*P* < 0.05 and ^##^*P* < 0.01 vs. FFA group

The levels of intracellular lipids and total glycerol (TG) were used in this study to evaluate the inhibitory effect of WE, WEE, and EE (750 *μ*g/mL) on FFA-induced intracellular lipid droplets and TG accumulation. The results showed that the TG and lipid levels significantly increased after FFA stimulation for 24 h (Figure 3, *P* < 0.01). WE, WEE, and EE all exhibited the ability to attenuate intracellular lipid and TG accumulation in LO2 hepatocytes induced by FFA (Figure 3). Treatment of LO2 cells with EE resulted in a significant lipid-regulating effect compared to the FFA group (*P* < 0.05). These results suggested that the 50% ethanol extraction of HGQZ could ameliorate lipid accumulation and TG enhancement to achieve a lipid-lowering effect.

### 3.5. Potential Q-Marker Prediction Based on Pearson Correlation Analysis

The relationship between the bioactivity and quality of these extracts was established by performing correlation analysis. The results showed that the contents of six chemical components, including typhaneoside, hyperoside, isoquercitrin, isorhamnetin-3-O-neohesperidoside, notoginsenoside R1, and alisol B 23-acetate, were positively correlated with the lipid-lowering effect of HGQZ (Table 2). These results indicated that these six components might be the potential active compounds of HGQZ.

### 3.6. Verification of Quality Markers in Free Fatty Acid-Induced LO2 Cells

**Figure 4:**
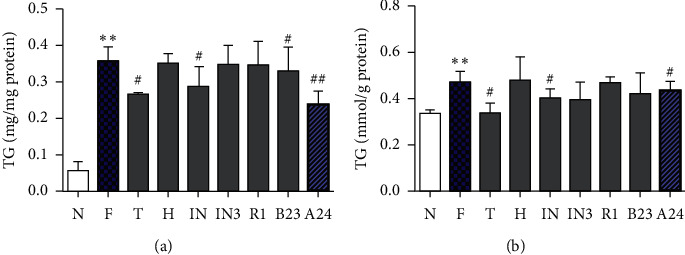
The effect of potential monomers on total glycerol (TG) and total cholesterol (TC) in the free fatty acid- (FFA-) induced LO2 cell model. N, control; F, FFA group; T, FFA and typhaneoside group (10 *μ*M); H, FFA and hyperoside group (10 *μ*M); IN, FFA and isoquercitrin (10 *μ*M); IN3, FFA and isorhamnetin-3-O-neohesperidoside group (10 *μ*M); R1, FFA and notoginsenoside R1 group (10 *µ*M); B23, FFA and alisol B 23-acetate group (10 *μ*M); A24, FFA and alisol A 24-acetate (10 *μ*M) group. The final concentration of FFA in each group was 1 mM. Data are presented as the mean ± SD of three independent experiments. *N* = 3. ^∗∗^*P* < 0.01 vs. control group; ^#^*P* < 0.05 vs. FFA group.

To validate the bioactivity of the potential active compounds, the effects of typhaneoside, hyperoside, isoquercitrin, isorhamnetin-3-O-neohesperidoside, notoginsenoside R1, and alisol B 23-acetate were evaluated in FFA-induced LO2 cells. Alisol A 24-acetate was chosen as a positive control. The results showed that the intracellular TC and TG levels of the typhaneoside, isoquercitrin, alisol B 23-acetate, and alisol A 24-acetate groups were all reduced compared with those of the FFA group (Figure 4).

## 4. Discussion

Previous studies have shown that the pathophysiology of NAFLD started from lipids in the liver. Lipids accumulate after lipid acquisition, and removal becomes imbalanced. Excess fatty acids that are decomposed by adipose tissue and flow into the liver are one of the causes of increased lipid contents in the liver [22]. In the present study, LO2 cells were exposed to FFA to simulate excessive FFA inflow into hepatocytes, resulting in hepatic steatosis. The lipid and TG contents in hepatocytes were considered typical indicators to investigate the lipid regulation effects of different extracts of HGQZ. Our study found that the 50% ethanol extraction of HGQZ could significantly attenuate the increased lipid and TG levels in the steatotic LO2 cells (*P* < 0.05).

As the extracts of HGQZ are complicated systems containing multiple components, it is very important to identify their chemical components. LC-QQQ-MS/MS analysis revealed that isoquercitrin, typhaneoside, isorhamnetin-3-O-neohesperidoside, and notoginsenoside R1 were the chemical components with high contents in these extracts. In addition, previous *in vivo* and *in vitro* studies have found that alisol B 23-acetate, alisol A 24-acetate, nuciferine, rutin, isoquercitrin, quercetin, isorhamnetin, and notoginsenoside R1 were effective in protecting against hepatic steatosis [23–29]. Therefore, these compounds may be identified as potential chemical markers, which may provide a chemical basis for the study of Q-markers in HGQZ.

In recent years, the combination of pharmacological and chemistry studies has offered an approach to explain the material foundation of the efficacy of TCM. In the present study, correlation analysis between the bioactivity and quality assessment showed that the contents of typhaneoside, hyperoside, isoquercitrin, isorhamnetin-3-O-neohesperidoside, notoginsenoside R1, and alisol B 23-acetate were positively correlated to the lipid-lowering effect (correlation coefficient >0.970). FFA-induced LO2 cells were used to verify the potential Q-markers predicted by the correlation analysis. The TC and TG levels in steatotic cells were reduced by the treatment of typhaneoside, isoquercitrin, alisol B 23-acetate, and alisol A 24-acetate after 24 h. The activity of typhaneoside (a flavonoid glycoside isolated from PT) towards the steatotic LO2 cells was estimated and validated for the first time.

At present, the quality control standards of Chinese medicines are mainly controlled by single-content and single-index methods. Although some methods use active ingredients as indicators, they are not necessarily the ones yielding all the functions of Chinese medicines. In this experiment, the medicinal materials were extracted with different solvents, and the contents of the index components in each extract were different. According to the different contents of the index components in the extract needed to change the effect of the drug, the Q-marker was screened. This experiment conducted Q-marker screening by specifying the different ingredients in the drug, which is a method that differs from the methods used by studies screening for Q-markers by identifying *in vivo* absorption changes. The advantage of this method was that the selected compounds were transparent and could be targeted for screening.

Based on the Q-marker concept [11], we considered typhaneoside, isoquercitrin, and alisol B 23-acetate as the Q-markers of HGQZ. First, these components are transferable and traceable in the process of the production and preparation of HGQZ formulas; second, they have been analyzed through either qualitative or quantitative methods; finally, the literature and our data have confirmed the effectiveness of the identified compounds in HGQZ. Therefore, typhaneoside, isoquercitrin, and alisol B 23-acetate are potential Q-markers for the quality control and research of HGQZ.

## 5. Conclusion

The identification of active compounds as quality markers is important for establishing a comprehensive and scientific quality control system for TCM. In this study, we analyzed the relationship between the bioactivity and chemical components of HGQZ for the first time. Extracts of HGQZ prepared by different solvents were applied to FFA-induced LO2 cells and compared. Simultaneously, the contents of twelve compounds with different high levels in three extracts were determined by LC-QQQ-MS/MS. We found that the contents of typhaneoside, hyperoside, isorhamnetin-3-O-neohesperidoside, notoginsenoside R1, and alisol B 23-acetate were positively correlated to the lipid-lowering efficiency of HGQZ. In addition, we verified the bioactivity of typhaneoside, isoquercitrin, and alisol B 23-acetate in an *in vitro* bioassay and identified them as Q-markers for HGQZ quality control.

## Figures and Tables

**Table 1:** The calibration curves, LOD, LOQ, precision, repeatability, stability, and recovery of 12 compounds.

Analyte	Linearity			LOD (ng/mL)	LOQ (ng/mL)	Precision RSD (%) (*n* = 6)	Repeatability RSD (%) (*n* = 6)	Stability RSD (%) (*n* = 6)	Recovery RSD (%) (*n* = 6)				
	Standard curves	*R* ^2^	Range (*µ*g/mL)						Original (*μ*g)	Spiked (*µ*g)	Found (g*µ*)	Recovery (%)	RSD (%)
S1	*y* = 933.45x + 103.94	0.9995	0.359∼11.5			1.68	1.51	1.91	2.95	2.87	5.82	99.79	3.05
S2	*y* = 590395x + 11861	0.9997	0.0833∼3.33	53.8	179	1.25	0.43	2.58	0.779	0.750	1.54	101.14	2.73
S3	*y* = 5979.1x + 1339.7	0.9991	0.870∼27.8	0.832	2.08	1.45	0.68	3.46	3.68	3.70	7.35	99.32	3.01
S4	*y* = 7174.5x + 191.03	0.9998	0.123∼4.92	21.7	43.5	1.93	1.16	3.84	0.263	0.369	0.626	98.32	2.06
S5	*y* = 4331.6x + 1160.2	0.9990	0.485∼15.5	18.4	61.5	0.97	0.69	2.18	6.41	6.05	12.4	100.82	2.60
S6	*y* = 11038x + 3741.7	0.9990	0.515∼10.3	12.1	24.2	1.84	0.28	3.35	1.08	1.24	2.29	98.47	2.20
S7	*y* = 9451.4x + 4995.3	0.9991	0.907∼14.5	25.7	128.7	1.11	2.12	3.01	7.25	6.65	13.7	97.53	1.15
S8	*y* = 1499.9x + 316.45	0.9990	0.518∼12.4	30.2	90.7	2.41	1.57	1.88	3.88	4.14	8.01	99.70	3.13
S9	*y* = 4893.2x + 731.86	0.9994	0.187∼2.24	155	259	3.48	2.81	2.00	0.453	0.355	0.806	99.52	3.77
S10	*y* = 2133.2x + 142.91	0.9997	0.0108∼0.260	14.0	46.7	2.80	2.33	3.02	0.336	0.303	0.638	99.65	3.46
S11	*y* = 682.62x−1.3265	0.9991	0.0310∼1.24	1.08	2.70	4.12	4.22	4.01	0.149	0.409	0.554	98.89	3.18
S12	*y* = 23163x−2708.8	0.9996	0.168∼6.72	0.310	0.620	1.90	1.16	4.23	2.47	2.24	4.67	98.43	1.38

**Table 2:** The qualification information of different extracts and correlation analysis results.

Analyte	Content (mg/g)			Pearson correlation coefficient
	WE	WEE	EE	
S1	0.004	0.007	0.146	0.915
S2	0.014	0.044	0.042	0.795
S3	0.278	0.352	0.459	1.000*∗*
S4	0.026	0.032	0.031	0.696
S5	0.252	0.337	0.414	0.993
S6	0.091	0.124	0.187	0.996
S7	0.148	0.207	0.348	0.989
S8	0.130	0.242	0.330	0.988
S9	0.026	0.067	0.070	0.849
S10	0.004	0.007	0.009	0.945
S11	0.010	0.018	0.054	0.964
S12	0.000	0.018	0.087	0.972

WE, water extract; WEE, water plus ethanol double extract; EE, 50% ethanol extract.

## Data Availability

The data used to support the findings of this study are included within the article.

## Supplementary Materials

Supplementary Materials Figure S1: full scan product ion mass spectra of the 12 compounds. Figure S2: cell viability assay on LO2 cells (WE, water extract; WEE, water plus ethanol double extract; EE, 50% ethanol extract; FFA, free fatty acid; cells were cultured in 96-well plates and treated with different doses of 50% ethanol extract for 24 hours; data are presented in mean ± SD of three independent experiments (*n* = 3); *∗∗P* < 0.01 vs. control group).
