# Supplementary material for: Discovery of Quality Markers in Hugan Qingzhi Formula by Integrating a Lipid-Lowering Bioassay with UHPLC-QQQ-MS/MS
Source: Evid Based Complement Alternat Med. 2020 Nov 12;2020:1594350. doi: 10.1155/2020/1594350 (PMC8860508; doi:10.1155/2020/1594350)
Supplement: Supplementary Materials — Figure S1: full scan product ion mass spectra of the 12 compounds. Figure S2: cell viability assay on LO2 cells (WE, water extract; WEE, water plus ethanol double extract; EE, 50% ethanol extract; FFA, free fatty acid; cells were cultured in 96-well plates and treated with different doses of 50% ethanol extract for 24 hours; data are presented in mean ± SD of three independent experiments (n = 3); ∗∗P < 0.01 vs. control group). [file 1594350.f1.docx]

**Supporting information for:**

**Discovery of Quality Markers in Hugan Qingzhi Formula by Integrating a Lipid-lowering Bioassay with UHPLC-QQQ-MS/MS**

**Fan He,^1, #^ Chun-Xin Xiao,^2, #^ Can-Jian Wang,^1, #^ Jie Liang,^1^ Qi-Qing Cheng,^1^ Li Zhang,^1^ Ben-Jie Zhou,^2, *^ and Hua Zhou ^1,3,4 *^**

^1^ State Key Laboratory of Quality Research in Chinese Medicine and Faculty of Chinese Medicine, Macau University of Science and Technology, Taipa, Macao, P.R. China

^2^ Department of Pharmacy, The Seventh Affiliated Hospital, Sun Yat-sen University, Shenzhen, P.R. China

^3^ Zhuhai Hospital of Integrated Traditional Chinese and Western Medicine, Zhuhai City, Guangdong Province 519000, P.R. China

^4^ Joint Laboratory for Translational Cancer Research of Chinese Medicine of the Ministry of Education of the People’s Republic of China, Macau University of Science and Technology, Taipa, Macao, P.R. China

* Correspondence should be addressed to Hua Zhou; hzhou@must.edu.mo and Benjie Zhou; zhoubj163@163.com

^#^ These authors contributed equally to this work and should be considered co-first authors.

**Supplementary caption**

Figure S1: Full scan product ion mass spectra of the 12 compounds.

Figure S2: Cell viability assay on LO2 cells. (WE, water extract; WEE, water plus ethanol double extract; EE, 50% ethanol extract; FFA, free fatty acid. Cells were cultured in 96-well plates and treated with different doses of 50% ethanol extract for 24 hours. Data are presented in mean ± SD of three independent experiments. n=3. ^∗∗^P<0.01 vs. control group.)


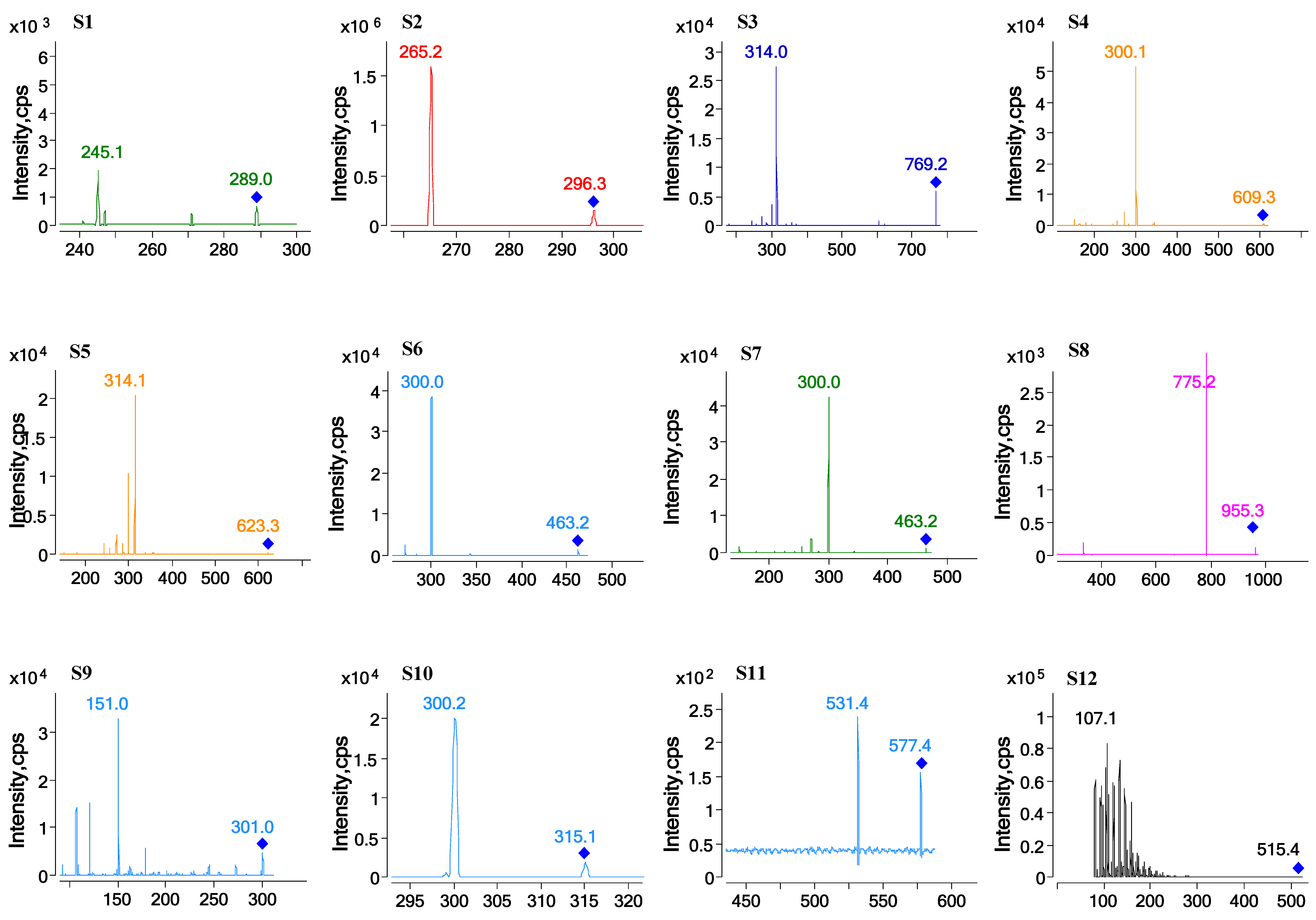


Figure S1: Full scan product ion mass spectra of the 12 compounds.

**
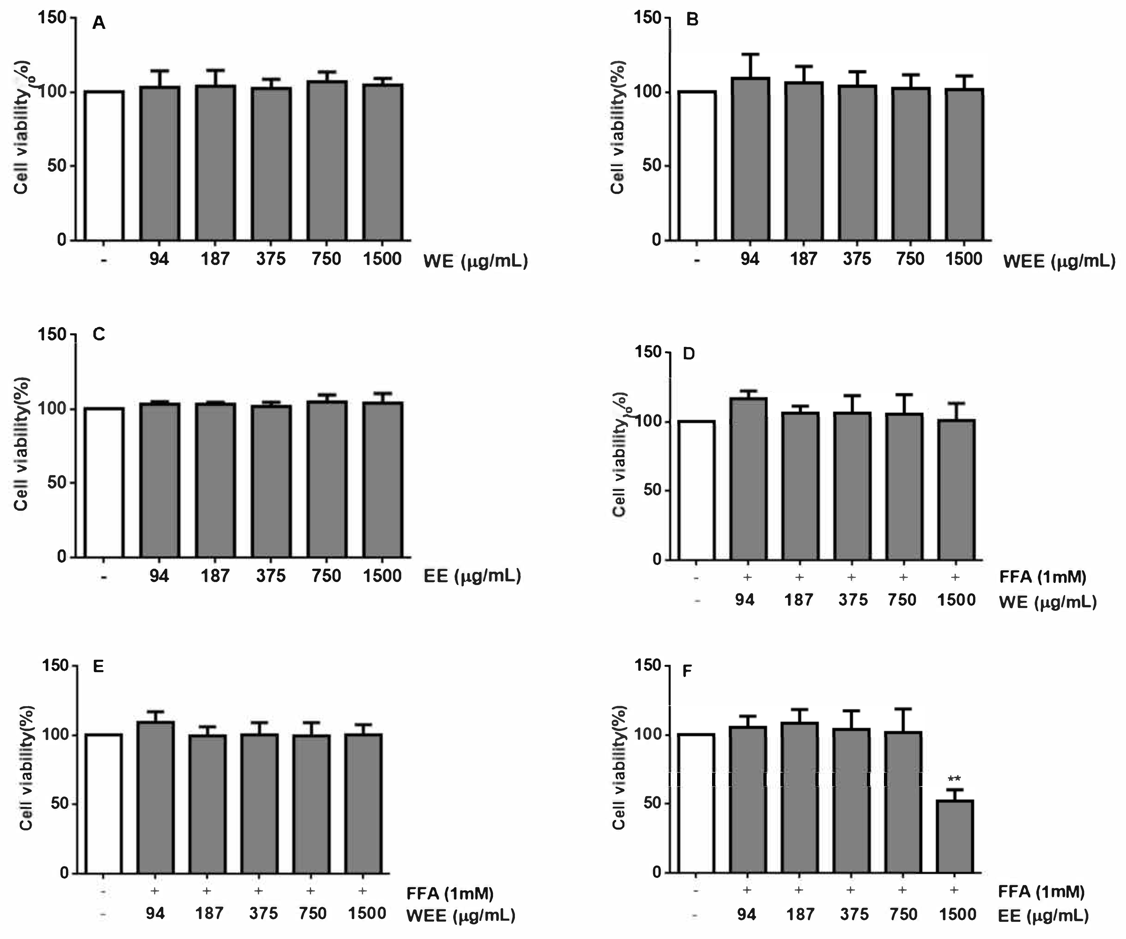
**

Figure S2: Cell viability assay on LO2 cells. (WE, water extract; WEE, water plus ethanol double extract; EE, 50% ethanol extract; FFA, free fatty acid. Cells were cultured in 96-well plates and treated with different doses of 50% ethanol extract for 24 hours. Data are presented in mean ± SD of three independent experiments. n=3. ^∗∗^P<0.01 vs. control group.)
